# Enhancing the notification system for surveillance of infectious diseases in Qatar during the FIFA World Cup 2022: project overview

**DOI:** 10.1186/s12889-024-18016-9

**Published:** 2024-02-27

**Authors:** Wafa Ibrahim, Sayed Himatt, Sara Heikal, Maha Al Shamali, Raihana Jabbar, Tasneem Elshareif, Agnes Bakiri, Montaha Mahmoud, Rula Shami, Hanaa Saeed, Ghaydah Al Shami, Douaa Omer, Esraa Barakat, Lylu Mahadoon, Taysser Elshaikh, Rania Rahma, Entesar Omer, Aiman Elbourdiny, Hamad Al Romaihi, Mohamed Al Thani, Mohamed Sallam

**Affiliations:** 1 Ministry of Public Health, Doha, Qatar; 2 School of Medicine, Cairo University, Cairo, Egypt; 3 College of Dental Medicine, Qatar University, Doha, Qatar

**Keywords:** Surveillance, Notification System, Infectious diseases, Reporting rate, FIFA World Cup 2022, Qatar

## Abstract

**Background:**

In 2022, the Surveillance Department of the Ministry of Public Health in Qatar adopted an integrated project called the Notification Enhancement Project (NEP) to enhance the infectious disease notification system. Efficient surveillance and notification promote early alerts and allow immediate interference in reducing morbidity and mortality from outbreaks. The project was designed to improve the knowledge, attitudes, practices, and notification processes of healthcare workers in Qatar by increasing their reporting rates.

**Methods:**

The strategy for comprehensively enhancing notifications was based on the observation and evaluation of the current notification system, the implementation of interventions, and post-evaluation follow-up. To implement the project, we relied on three aspects: effective methods used in previous relevant studies through a literature review, feedback received from healthcare workers, and suggestions from public health surveillance experts from the Ministry of Public Health, Qatar. A preassessment was conducted through an online survey by the Ministry of Public Health. The effectiveness of the different interventions was assessed by analyzing the data of notified patients reported through the Disease Surveillance and Reporting Electronic System. Pre- and postintervention assessments were performed by comparing the percentage of patients notified by healthcare providers with that of patients confirmed by healthcare providers in the laboratory to compare the notification rates over three time periods between January and December 2022.

**Results:**

There was significant improvement in the infectious disease notification process. A comparison before and after the implementation of the interventions revealed an increase in the communicable disease notification rate among healthcare workers. Pre- and postintervention data were compared. Infectious disease notification activities by healthcare workers increased from 2.5% between January and May 2022 to 41.4% between November and December 2022.

**Conclusion:**

This study highlights the efficiency of different interventions in correcting the underreporting of infectious diseases. Our findings suggest that implementing the Notification Enhancement Project significantly improves notification rates. We recommend continuing interventions through constant education and training, maintaining solid communication with HCWs through regular reminder emails and feedback, periodic assessment of the electronic notification system, and engagement of healthcare workers and other stakeholders to sustain and expand progress achieved through continuous evaluation.

**Supplementary Information:**

The online version contains supplementary material available at 10.1186/s12889-024-18016-9.

## Introduction and background

Surveillance of infectious diseases is a critical epidemiological tool for monitoring and keeping track of the health of the population [1]. The three main surveillance objectives include describing the epidemiology and presentation of the disease burden, observing disease trends, and identifying novel pathogens and outbreaks [1]. This process is achieved by systematically collecting, analyzing, and interpreting disease data and public health-related events [2, 3]. Timely notification of infectious diseases using established reporting tools is crucial [4]. Several diseases are mandated by law to be notified to governments and public health authorities; these diseases are classified as notifiable diseases [5, 6]. Making a disease legally ‘notifiable’ allows intervention to control the spread of highly infectious diseases. In less infectious conditions, this approach improves the understanding of the burden and distribution of the disease [7, 8].

A vital indicator of an effective surveillance system is the speed between the steps from receiving notification until an action or decision is made [9]. Delays in reporting infectious diseases can hamper prompt outbreak control [10]. As a result, Qatar mandated by law that every healthcare practitioner and laboratory should report infectious diseases [11]. Surveillance system enhancement helps in the timely containment of the spread of infectious diseases [12]. The enhancement of surveillance systems falls into different categories, starting from improving the mandated reporting of cases, establishing automated linkages between healthcare information systems and public health systems, and training healthcare personnel to use the systems effectively [12, 13].

Because mass gatherings pose many health risks to the public, the Surveillance and Outbreak Response section at the MOPH took many initiatives to enhance its disease detection processes. In preparation for the hosted FIFA Arab Cup 2021 and FIFA World Cup 2022 tournaments held in Qatar, the Ministry of Public Health (MOPH) established a national electronic surveillance and vaccine registry system called the Surveillance and Vaccination Electronic System (SAVES). The goal of the system is to provide a platform where all national healthcare facilities, hospitals, and laboratories can notify decision-makers of infectious diseases, keep records of vaccines taken by community members, facilitate the retrieval of patient information, and produce data-driven reports of daily and weekly trends in high-priority diseases.

The actual implementation and testing of SAVES began in early 2020, during the COVID-19 pandemic. It has been heavily utilized for COVID-19 investigations and vaccination records and as a key surveillance tool for the Arab FIFA Cup 2021. The adoption of SAVES was one of the greatest additions to the national healthcare system and has safeguarded the health of many citizens and residents, especially during the pandemic. However, these systems have limitations that need to be improved to improve surveillance of communicable diseases. Therefore, as part of the preparations for the FIFA World Cup Qatar 2022, a wide range of enhancements were made to maximize the aim of SAVES and strengthen the system.

In early 2022, the surveillance and outbreak response section at the MOPH developed the Notification Enhancement Project (NEP). The goal of the project was to enhance the communicable disease notification system in Qatar based on the challenges identified in the system during the COVID-19 pandemic and the FIFA Arab CUP 2021 (FAC21) experience, with a focus on enhancing the knowledge, attitudes, and practices of healthcare workers in notifying communicable diseases, notification processes and surveillance tools and establishing a solid communication channel with healthcare workers.

Various interventions have been adopted to implement this enhancement process based on the effective methods employed in the previous relevant literature and those suggested by Qatar’s public health surveillance experts. To the best of our knowledge, no previous studies have described surveillance enhancements made in preparation for mass gatherings using the same methods.

## Methods

**Fig. 1:**
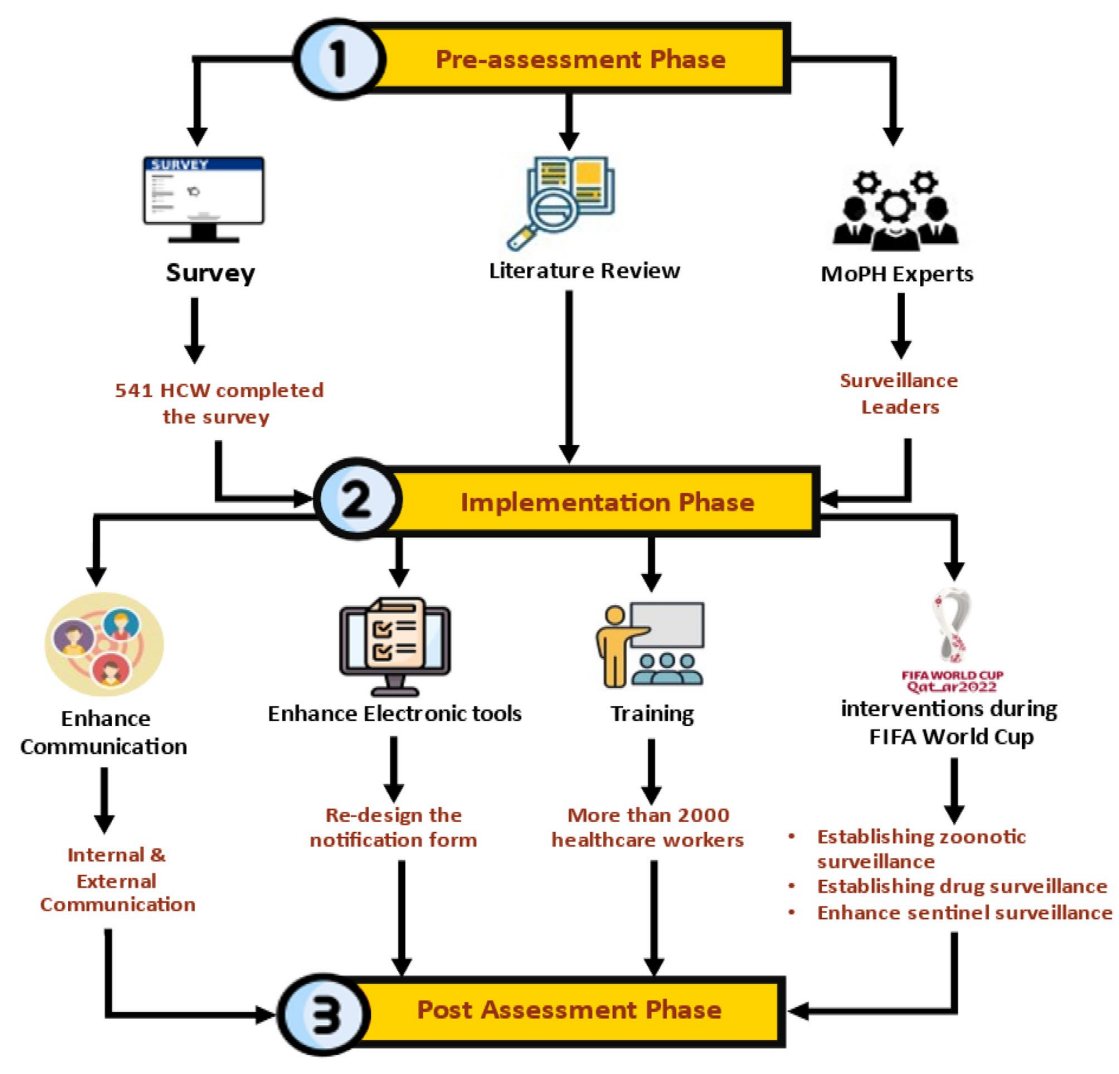
This Figure shows the Notification enhancement project flowchart

The standard for comprehensive evaluation of the notification system was based on the use of a monitoring framework to evaluate the effectiveness of different interventions to improve notification at the national level from January 2022 to December 2022. The evaluation and enhancement of the notification system involved three phases (Fig. 1): (i) observation and evaluation of the current notification system, (ii) implementation of an intervention to improve notification, and (iii) post-evaluation and follow-up.

### Phase 1: Observation and evaluation: preassessment evaluation (1 January to 30 May 2022)

The first step was to perform a literature review of the enhancement of notification systems. All available data were reviewed and analyzed, focusing on the challenges associated with the infectious disease notification process, factors influencing proper notification implementation, and interventions performed to enhance notification.

#### HCWs’ knowledge, perspectives, and practices related to communicable disease notifications in Qatar

Preassessment survey: An online survey through the Ministry of Public Health (MOPH) was conducted to assess healthcare providers’ knowledge and awareness of the infectious disease notification system and determine the factors influencing the proper implementation of notification procedures. This included all healthcare workers in Qatar, including those in public and private sectors. The questionnaire comprised three parts: The first part contained questions concerning healthcare workers’ sociodemographic information and practice-related questions. The second part contained specific questions on infectious disease knowledge and practices. The third part contained specific questions about challenges, notification process satisfaction, and HCWs feedback and comments. In this phase, we formulated the most important recommendations and interventions that may help improve the notification process, including raising awareness of notification through regular surveillance and disease notification training and enhancing communication and engagement of HCWs.

#### Interventions suggested by the surveillance section supervisors in the MOPH*

The interventions suggested by MOPH experts were summarized as follows: creating a shorter list of notifiable diseases; conducting basic and refresher training programs on notification systems targeted at HCWs; setting focal points in all healthcare facilities in Qatar to create communication channels; establishing drugs; managing zoonotic diseases; and providing environmental surveillance.

#### Effective methods used in previous relevant studies

All related studies were reviewed using all the available keywords to enhance the notification system. After the literature review, we found that many studies showed that various approaches could improve notification rates, including training health professionals, implementing workshops, enhancing institutional electronic reporting systems, and engaging with and providing feedback to physicians [13–21].

### Phase 2: implementation phase (1 Jun– 31 Oct 2022)

To implement the Notification Enhancement Project (NEP), we relied on effective methods utilized in previous literature, feedback from healthcare workers, and feedback suggested by public health surveillance experts in the Ministry of Public Health, Qatar. The interventions performed (the different types of interventions are listed in Table 1.

**Table 1:** Types of interventions performed to enhance the notification

Type of interventions	Objective	Implementations/impact
Educational &Training	To enhance healthcare workers’ awareness about the importance of notifying diseases and their responsibility to report.	A-Performed online and on-site training for HCW (11 sessions in collaboration with governmental and private hospitals and departments inside MOPH) for HCW and Trained more than 2000 HCW
		b-Circulated training materials to HCW (Includes protocols, Alerts, Videos, posters, and leaflets)
		c-Participated in a workshop organized by the IPC (infection prevention and control department) team in MOPH
		d-Trained focal points in private and governmental facilities
		e-Trained the HCW in FIFA health facilities sites (FIFA clinic in stadiums and Fan zones)
Enhance notification tools	To make the process less time-consuming (as the main barrier to notifying is the high workload)	a-Enhanced the electronic notification system by redesigning and simplifying the notification form (Decreasing the number of required fields
		b-Assess the electronic system (SAVES)& the capacity of the software and Request for system upgrade based on the situation
Communication	-To engage influencers and decision-makers among healthcare workers to be part of the enhancement -To reinforce the importance of participating in public health surveillance.	Performed regular Email reminders through CDC email (to circulate Alert-protocols
		Notification Awareness campaign performed.
		Created an internal communication mechanism and workflow
		Strengthen the external communication pathway with healthcare facilities.
Interventions during FIFA WC	1) -To increase healthcare workers’ compliance for notification of notifiable diseases during the FIFA World Cup Qatar 2022	2) Strengthen the sentinel surveillance during the FIFA World Cup by increasing the number of HC facilities reporting to MOPH. (From 35 HC facilities to include all HC facilities during the FIFA World Cup)
		establishing drug surveillance during the FIFA World Cup
		4) Establishing zoonotic disease surveillance during the FIFA World Cup

This table shows the different interventions MOPH performed during 2022 to enhance the notification system

The proposed (NEP) interventions mainly focus on three areas:

- Training & Education: Enhancing healthcare workers’ knowledge, attitudes, and practices. Awareness of the importance of notifying infectious diseases, reporting requirements, and practice should be promoted.
- Enhance notification tools: Facilitate the notification process for healthcare workers and assess the current system’s capacity and the need to upgrade the system.
  The notification form was altered by decreasing the number of required fields. While maintaining the initial design due to the intricacy of change requests, the mandatory fields were reduced from 11 to 6, with the addition of two crucial fields. Consequently, the total number of remaining fields was reduced to eight. Because of these modifications, the time required for clinicians to complete the form was reduced. (Note: The list of notifiable diseases could not be modified because additional studies are needed to determine which diseases should be removed and kept on the list.) (see Table 1).
- Enhance communication: Engaging healthcare workers, stakeholders, and leaders in the notification process and establishing a solid communication channel between the MOPH, specifically the surveillance section, and healthcare workers in private and public health organizations.

Moreover, additional steps were taken to improve the notification system during FIFA WC 2022. Enhancing sentinel surveillance, establishing a zoonotic disease surveillance system, and Establishing drug surveillance.

Our interventions did not involve telephone calls but mainly depended on regular reminders through CDC mail.

### Phase 3: post-evaluation and follow-up phase

The impact of the interventions on improving the notification system was assessed by analyzing the data of notified patients reported through the SAVES (which is a disease surveillance and reporting system that allows sources (government/semi or private) to inform potential disease incidents, report communicable diseases and outbreaks, or declare cases of disease). Pre- and postintervention assessments were performed by comparing the rate of notified cases by healthcare providers with confirmed cases in the laboratory to compare the notification rates over three periods: Pre-NEP implementation (1st January to 31st May 2022), Post-NEP implementation (1st June to 31st October 2022), and during the event (FIFA World Cup 2022) from 1st November to 31st December.

The assessment was conducted as follows.

- before intervention: Data dated from January 1, 2022, to May 31, 2022.
- of SAVES data, number of notified cases from labs vs. cases reported by HCWs, and number of notified cases from the governmental sector vs. cases reported by the private sector.
- Preassessment assessment (after interventions): Data from June 1 to October 31, 2022.
  Analysis of SAVES data, number of notified cases from labs vs. cases reported by HCWs, and number of notified cases from the governmental sector vs. cases reported by the private sector.
- Post assessment (during the FIFA World Cup 2022): Data from 1st November to December 31, 2022; analysis of SAVES data; number of notified cases from labs vs. cases notified by HCWs; and number of notified cases from the governmental sector vs. cases notified by the private sector.
- Hypothesis Testing: Using the paired t-test for the difference in means of pre- and post-notifications, the statistical significance has been calculated with 95% Confidence.
- The differences in the percent of notifications during the three phases were computed.

#### The impact of the notification enhancement project (NEP) on enhancing the communicable diseases surveillance system

## Results

Results of the preassessment survey: The survey collected information about the sociodemographic and practice-related characteristics of healthcare workers and assessed HCWs’ knowledge of surveillance and reporting practices. A total of 2000 questionnaires were distributed online through CDC emails to all eligible HCWs, and 541 HCWs responded to the survey. The data showed that most healthcare workers were aware of the importance of timely notification of communicable diseases. The study revealed that the major factors that influenced proper implementation of the notification procedure were a high workload (58%), not receiving feedback information (47.3%), inadequate training on reporting diseases (45.3%), reluctance to violate patient privacy (41%), the time required to complete the notification procedure (36%), and unclear notification procedures (25%). Therefore, the factors contributing to the underreporting of infectious diseases were summarized as a lack of knowledge, lack of feedback and communication, and complicated notification processes. The interventions were then planned and classified into three main categories: training, enhanced notification tools, and communication.

Results of pre- and postintervention assessments (analysis of SAVES data): An increase of 27% (from 2.5 to 29%) was noted in the proportion of notifications received from the HCWs. Interventions that enhanced reports using different techniques improved the reporting of communicable diseases. The highest increases in total notifications by healthcare workers were noted for SARIs, scarlet fever, and meningitis. A 214% increase in total reports from healthcare workers was noted when comparing the pre-and postintervention periods.

The analysis showed that the number of postintervention notifications was significantly greater than that in the preintervention phase. Communicable disease notification activities by healthcare workers increased from 2.5% between January and May 2022 to 29% between June and October 2022 and reached 41.4% between November and December 2022. (Table 2, Appendix A, B, C).

**Table 2:** Notification rates received over three time periods (from 1 January to 31 December 2022)

Periods	Government sectors		Private sectors		Total	
	Notifications received from Lab	Notifications received from HCWs	Notifications received from Lab	Notifications received from HCWs	Notifications received from Lab	Notifications received from HCWs
Preintervention (1 Jan– 31 May 2022)	97.4%	1.2%	0.1%	1.3%	97.5%	2.5%
Post-intervention 1 (1 Jun– 31 Oct 2022)	70.7%	19.2%	0.3%	9.9%	71%	29%
Post-intervention 2 (1 Nov– 31 Dec 2022)	58.1%	27.3%	0.4%	14.1%	58.5%	41.4%

**Fig. 2:**
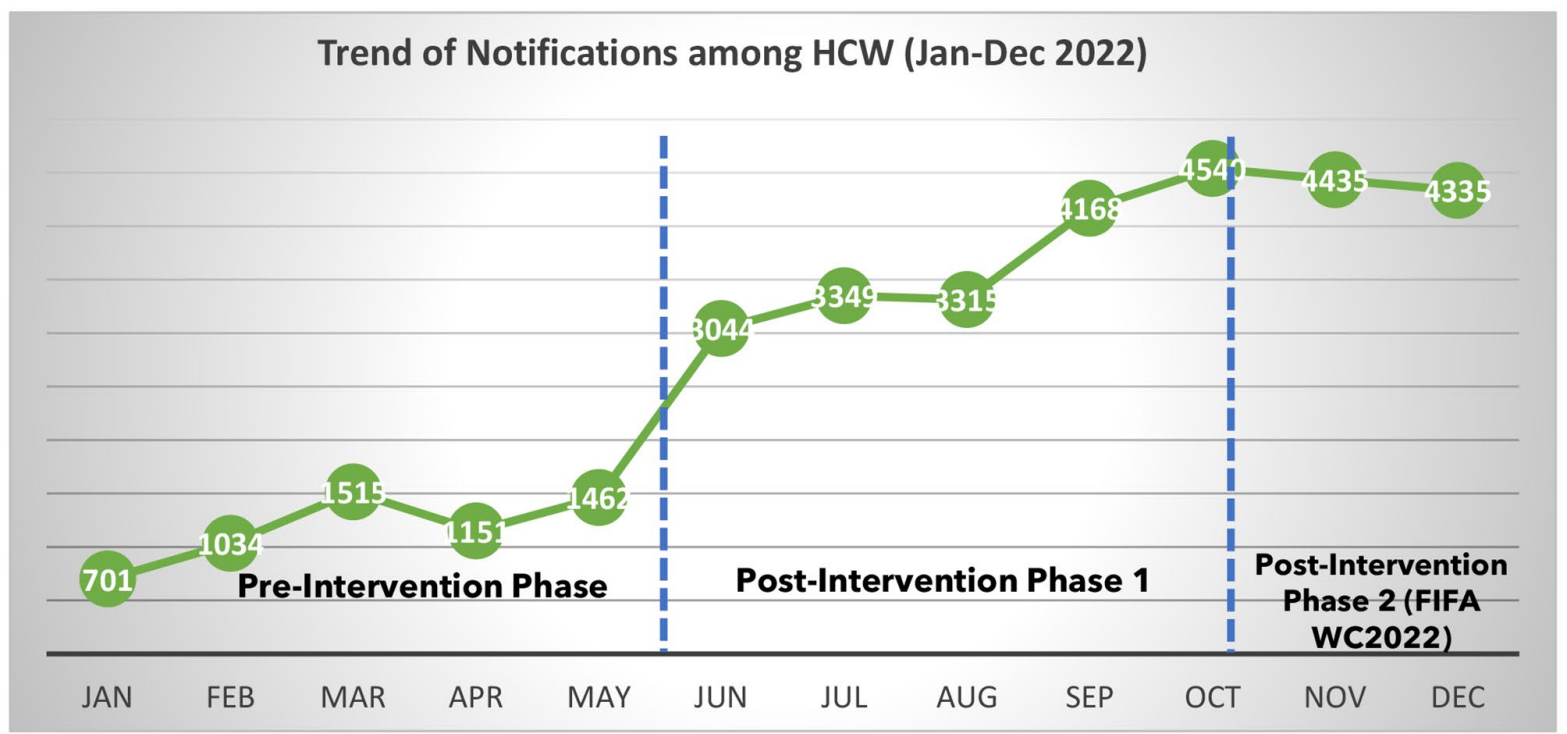
Trend of notification by HCWs from 1st Jan to 31st Dec 2022

Our results show an increase in the proportion of notifications by HCWs throughout the year and a prominent increase in the proportion of reports by HCWs by the third quarter of the year. As shown in Fig. 2.

### Monthly notification lab vs. HCWs

**Fig. 3:**
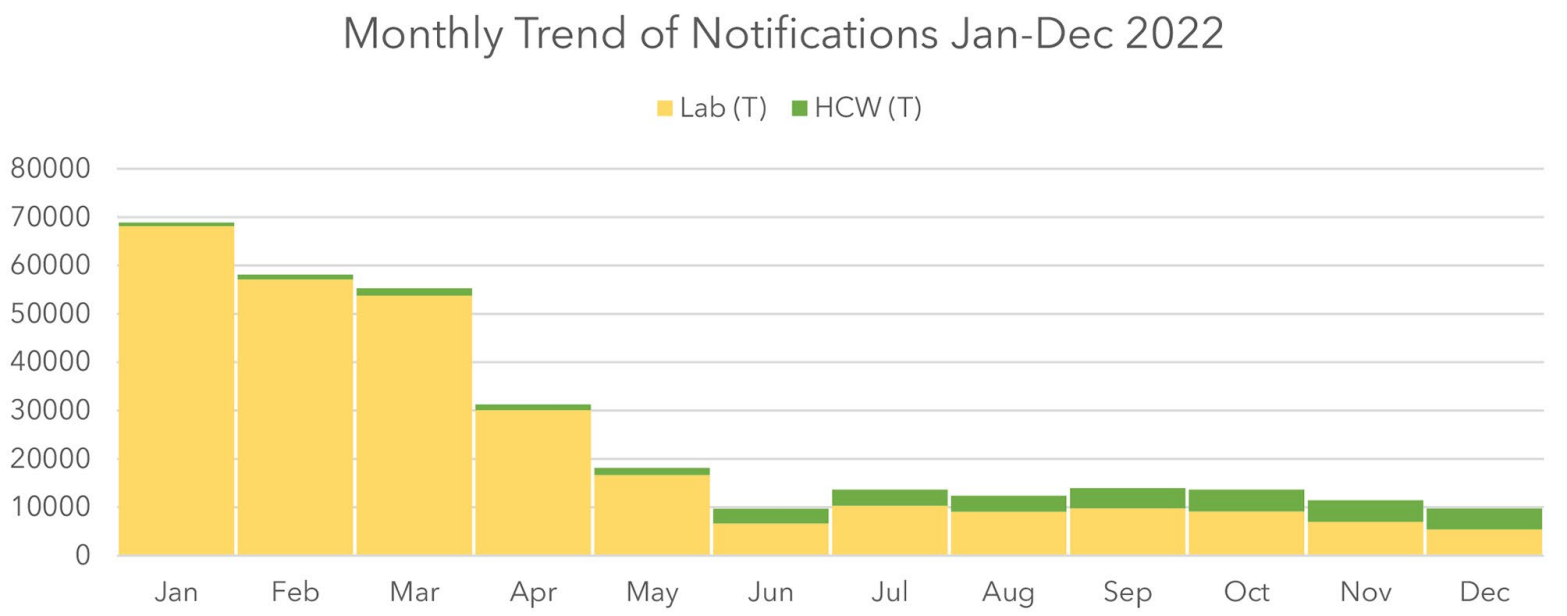
This Figure shows the trend of notification rate from January to December 2022: a monthly comparison between lab and HCWs notifications

The following graph shows a monthly comparison between laboratory and HCW notifications. The total notifications show a decreasing trend in the year’s first two quarters and maintain a steady trend after that. The proportion of reports from HCWs increased by the year’s third quarter (Fig. 3). The overall decrease in notifications could be attributed to the reduction in the number of COVID-19 cases reported after the Omicron wave. Nevertheless, an increase in notifications among HCWs can be observed.

## Discussion

This study highlights the efficiency of different interventions in correcting the underreporting of notifiable infectious diseases in Qatar. Our findings suggest that implementing interventions, for instance, by enhancing knowledge and training, enhancing reporting tools, and enhancing communication projects, was significantly associated with an enhanced notification rate and improved notifications and surveillance systems.

By the third quarter of the year, during the FIFA WC in Qatar in 2022, the interventions positively impacted healthcare workers’ compliance with the notification process for notifiable diseases. The increase in notification rates among HCWs is evident by comparing the preintervention and postintervention phases. The difference in laboratory notification rates might be explained by the decrease in COVID-19 incidence during this period.

These findings are consistent with the findings of many studies in which notification rates could be improved through various approaches, including training health professionals, implementing workshops, emphasizing the importance of the process for notifiable diseases, enhancing institutional electronic reporting systems; engaging with and providing feedback to physicians may also help improve reporting rates [13, 14]. For instance, a study conducted in Uganda discussed the role of training healthcare providers in enhancing notifications and showed improved neonatal data capture after training and mentorship [14]. A similar study conducted in Peru showed that the rate of reporting on time significantly increased after training sessions were provided to healthcare workers [15]. Two relevant studies related to the FIFA World Cup in Germany in 2006 and 2011 discussed enhancing the notification system by making mandatory daily notifications instead of weekly notifications, thereby accelerating data transmission [16, 17]. Another study introduced a new electronic system to enhance the surveillance system in Kenya (a mobile SMS-based disease outbreak alert system) to report immediately notifiable diseases within 24 h. With this system, notification and response rates have increased [18]. Another study was conducted in China and Madagascar, where the sentinel surveillance strategy was employed to ensure early detection and timely isolation of suspected cases [19, 20]. Another study revealed that multisectoral collaboration might help strengthen the disease surveillance system in humans and animals [21].

This study focused not only on identifying the challenges but also on finding solutions by collecting all the available results and solutions. The results of this study can be used as a reference by researchers and public health professionals. This approach can provide relevant information to determine methods and strategies for increasing the notification rate and enhancing local and global surveillance systems for communicable diseases.

### Research recommendations

Continuing evaluation and improvement by conducting additional research is needed to assess notification systems for the surveillance of communicable diseases to find ways to improve national and international surveillance systems. Follow-up questionnaires will be mailed through CDC email, and the data will be analyzed to assess the notification status and progress in the system.

## Conclusion

In conclusion, different interventions improved the notification rate overall and significantly affected the number of cases reported. The intervention played an important role in simplifying the reporting tools by redesigning the electronic notification form, raising awareness about notifications of communicable diseases through continuous training and educational sessions, and providing regular reminder emails to HCWs. Moreover, the interventions played an important role in improving communication between the Ministry of Public Health and the healthcare facilities in Qatar. Our interventions did not involve telephone calls but were primarily regular reminders sent through CDC mail. We recommend continuing the evaluation and enhancement of surveillance system measurements.

### Electronic supplementary material

Below is the link to the electronic supplementary material.

Supplementary Material 1

## Data Availability

Data that supports the findings will be provided by the corresponding author (Dr. Mohamed Sallam) upon request.

## Abbreviations

HCWs: Healthcare workers
MOPH: Ministry of Public Health
NEP: Notification Enhancement Project
SAVES: Surveillance and Vaccination Electronic System, in Qatar
