# Supplementary material for: Enhancing the notification system for surveillance of infectious diseases in Qatar during the FIFA World Cup 2022: project overview
Source: BMC Public Health. 2024 Feb 27;24:625. doi: 10.1186/s12889-024-18016-9 (PMC10900677; doi:10.1186/s12889-024-18016-9)
Supplement: Supplementary file 1 — Supplementary Material 1 [file 12889_2024_18016_MOESM1_ESM.docx]

**Appendix**
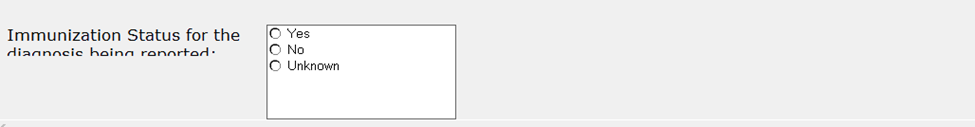


## **The proportion of lab notifications vs. HCW notifications preintervention and postintervention**

**Appendix A: HCW notifications out of the total notifications 2.5% preintervention (pre-intervention)**

**Appendix B: An increase in the proportion of notifications received from HCW from 2.5% to 29% in postintervention 1.**

**Appendix C: The proportion of notifications received from HCW increased from 29% to 41.4% in postintervention 2**.
